# Effect of Initial Orientation on the Anisotropy in Microstructure and Mechanical Properties of 2195 Al–Li Alloy Sheet during Hot Tensile Deformation

**DOI:** 10.3390/ma16145012

**Published:** 2023-07-15

**Authors:** Jian Ning, Jiangkai Liang, Xinyu Hu, Xianggang Ruan, Zhubin He

**Affiliations:** State Key Laboratory of High-Performance Precision Manufacturing, School of Mechanical Engineering, Dalian University of Technology, Dalian 116024, China; ningjiannj@126.com (J.N.); 15535368969@163.com (J.L.); huxy085@163.com (X.H.); ruanxg530@163.com (X.R.)

**Keywords:** 2195 Al–Li alloy, hot tensile test, anisotropy, dynamic recrystallization, microstructural evolution

## Abstract

The 2195 Al–Li alloy, as one of the representative third-generation Al–Li alloys, has extensive applications in lightweight aerospace structures. In this paper, the anisotropy in mechanical properties and microstructure evolution of 2195 Al–Li alloy sheets were investigated under a strain rate of 0.01, 0.1, 1 s^−1^ and a temperature of 440 and 500 °C. Experimental results showed that the hot tensile properties of the 2195 Al–Li alloy sheet exhibited a strong dependence on loading directions. The peak stress (PS) and elongation (EL) along the rolling direction (RD) were larger than the transverse direction (TD). For the tests carried out at 440 °C-1 s^−1^, the PS values of the sheets stretched along the RD and TD are 142.9 MPa and 110.2 MPa, respectively. And, most of the PS anisotropy values are larger than 15%. The anisotropy in EL is less significant than in PS. All the differences are about 10%. Moreover, dimples in the samples stretched along RD were more and deeper than those along TD at 440 °C. The fracture morphology along RD and TD were similar, and both were cleavage fractures at 500 °C. Particularly, the fractions of high angle grain boundaries (HAGBs) along TD were all about 5% larger than those of RD. And, there were more small-sized continuous dynamic recrystallization (CDRX) grains inside the initial grains and discontinuous dynamic recrystallization (DDRX) grains featured with the local bulge of grain boundaries along TD. This was due to the smaller average Schmid factor and the vertical EL trend of the initial grains when the samples were stretched along TD. A model of grain evolution during the dynamic recrystallization (DRX) along RD and TD was proposed based on EBSD results. The Schmid factor and banded structure had a more prominent effect on the hot ductility of the 2195 Al–Li alloy compared with the degree of DRX, thus presenting a higher EL and better hot ductility along RD.

## 1. Introduction

Al–Li alloys are a new type of Al alloy with Li as one of the major alloying elements. It has excellent mechanical properties of high strength and high specific stiffness, suitable physicochemistry properties of low density, outstanding corrosion resistance and heat resistance, and processing properties that can be enhanced through heat treatment [1,2,3,4]. Experimental research shows that the density of the alloy can decrease by 3%, and the elastic modulus can increase by 6% for each 1% Li added to Al [5,6]. The above excellent performance improvements led to extensive applications of Al–Li alloys in the aviation area. From the late 1980s to the early 1990s, the third generation of Al–Li alloys appeared and began to be substantively used in the aerospace field. Among them, the most representative ones are the 1460 (Al–Cu–Li) and 2195 (Al–Cu–Li) Al–Li alloys used in spacecraft fuel tanks [7].

The 2195 Al–Li alloy is a typical third-generation Al–Li alloy in the application of spacecraft fuel tanks. The use of the 2195 Al–Li alloy enables a reduction in fuel tank weight and an increase in strength. In previous studies, the tensile strength of the second-generation aerospace Al alloy material 2219 (Al–Cu)-T8 sheet can reach 466 MPa. In comparison, the value of 2195-T8 can reach 556 MPa. Moreover, fuel tanks composed of the 2195 Al–Li alloy could offer better reliability due to the lower fatigue crack expansion rates and better low-temperature performance [8,9]. In recent years, the hot stamping process has been applied to form Al alloy sheets to obtain Al alloy parts with complex shapes and high strength and solve the problem of low formability at room temperature [10]. On this basis, Yuan et al. [11] applied the hot stamping process to a 2195 Al–Li alloy sheet to solve the problems of easy cracking, high resilience, and large heat treatment distortion in forming rocket fuel tank parts at room temperature. However, the current hot forming of the 2195 Al–Li alloy sheet is still in the research stage, and more basic research on the hot deformation macro- and micro-mechanism of the 2195 Al–Li alloy sheet is necessary to be carried out, which can provide a more experimental insight for practical application in the aerospace field.

Anisotropy, also known as “heterogeneity”, refers to the fact that the physical and chemical properties of the alloy show certain variabilities along different loading directions; that is, the performances of the alloy obtained in different directions are different. Previous studies showed that anisotropy has an obvious effect on the forming performance of the sheet; it may cause defects such as tearing and cracking in the stamping process and reduce the forming limit of the material [12]. Compared with the traditional Al alloy, the anisotropy of Al–Li alloy is more significant. It is mainly caused by the strong interaction between the precipitations of the Al–Li alloy and the higher degree of deformation texture [13,14]. Therefore, some researchers have carried out studies on the anisotropy of Al–Li alloy sheets to analyze the influence of different process parameters on its anisotropy and the related microstructural mechanism. Wang et al. [15] studied the precipitation and texture-forming mechanism of the 2A97 (Al–Cu–Li) Al–Li alloy, and the influence of the precipitation and texture on the anisotropy was also analyzed. El-Aty et al. [16] investigated the influence of specimen orientation and the strain rate on the tensile performances and anisotropy mechanism of 1460, 8090 (Al–Li–Cu–Mg), and 2060 (Al–Cu–Li) Al–Li alloy sheets through dynamic and quasistatic tensile tests. Zhao et al. [17] studied the effects of different heat treatment processes on the anisotropy of the 2198 (Al–Cu–Li) Al–Li alloy and established the anisotropic yield stress analytical model of the material under different heat treatment and pre-deformation conditions. Zhao et al. [18] studied the influences of extrusion temperature and deformation rate on the grain structure and mechanical properties of the 2195 Al–Li alloy in different deformation directions. Peng et al. [19] studied the effect of grain orientation on creep aging behavior and microstructural evolution of 2195 Al–Li alloy plates under different stress levels at 180 °C and established a constitutive model considering the anisotropy of creep deformation and aging strengthening. These studies analyzed the processing parameters–microstructure–anisotropy linkages of the Al–Li alloy and provided a guide for optimizing the forming process of the Al–Li alloy under room or warm temperature conditions.

At present, the research on the hot stamping process of the 2195 Al–Li alloy has been extensively carried out. These studies mainly emphasize the structure and performance changes of the 2195 Al–Li alloy under typical forming methods and conditions. And, the effect of deformation parameters such as temperature on the mechanical properties and microstructure of the 2195 Al–Li alloy sheet, as well as the evolution of grain and substructure with increasing strains, are reported [20,21,22]. However, similar to the room temperature stamping deformation, the anisotropy can significantly affect the hot forming performance, causing problems such as differences in grain structure, uneven rheology, and even local cracking for the actual production processing [11,23]. Therefore, it is important to study the anisotropy of the 2195 Al–Li alloy under high-temperature deformation conditions. However, the present studies rarely involved the anisotropy of the 2195 Al–Li alloy under hot tensile deformation conditions.

In the current work, hot tensile tests, optical microscope (OM), scanning electron microscope (SEM), electron backscatter diffraction (EBSD), and transmission electron microscopy (TEM) studies were carried out to investigate the anisotropy in hot tensile deformation mechanic properties and microstructure evolution regularity of the 2195 Al–Li alloy. The discussion on microstructure mainly focused on grain and dislocation structure evolution in the process of dynamic recovery (DRV) and dynamic recrystallization (DRX) and their relations with the anisotropy in mechanical properties. The research enables the provision of a new experimental understanding of the mechanical mechanism and microstructure evolution for the 2195 Al–Li alloy rolled sheets in the progress of hot tensile deformation and further improves the processing quality.

## 2. Experimentation

### Material and Methods

The 2195 Al–Li alloy was studied with its chemical element composition detailed in Table 1. Cylindrical samples with the size of φ 4 × 110 mm were sliced from the sheet with an initial thickness of 8 mm. Hot tensile tests were conducted at 440 °C and 500 °C, and strain rates of 0.01, 0.1, and 1 s^−1^ on a Gleeble-3800 thermal simulation machine along rolling direction (RD) and transverse direction (TD). The samples were heated to the desired temperature and held for 600 s. The heating was performed at 5 °C s^−1^ from ambient temperature and then stretched to failure or certain strains.

The specimen for initial microstructure testing was cut from the initial 2195 Al–Li rolled sheet center. OM observations were performed on the ZEISS-K400 metalloscope (ZEISS company, Oberkochen, Germany). The fracture morphology was examined by SEM. The samples for microstructural examination were cut from the center of the fracture location after the tensile tests. The grain structure characterizations were conducted by EBSD in a Sigma 500/VP scanning electron microscope (ZEISS company, Oberkochen, Germany). The analysis of EBSD results, including the orientation, was performed in the Channel 5 EBSD system. The samples for EBSD studies were machined and then polished mechanically. The following electrochemical polishing was carried out at 16 V for 40 s in the electrolytical solution of 10% vol. perchloric acid and 90% vol. alcohol. The morphology of dislocations and grains was studied employing a Tecnai-G220 field emission transmission electron microscope (FEI company, Hillsboro, OR, USA). The TEM samples were ground into foils until the thickness was 0.04 mm, then twin-jet electro-polishing was carried out employing the solution of 70 vol % methanol and 30 vol % nitric acid at approximately −20 °C and 20 V.

## 3. Results and Discussion

### 3.1. Flow Behavior and Mechanical Anisotropy

**Figure 1:**
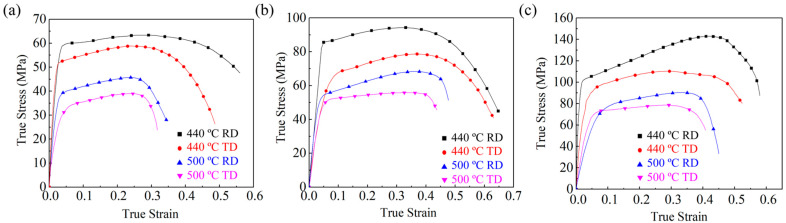
True stress–true strain curves of the 2195 Al–Li alloy at different strain rates of (**a**) 0.01 s^−1^, (**b**) 0.1 s^−1^, and (**c**) 1 s^−1^.

**Figure 2:**
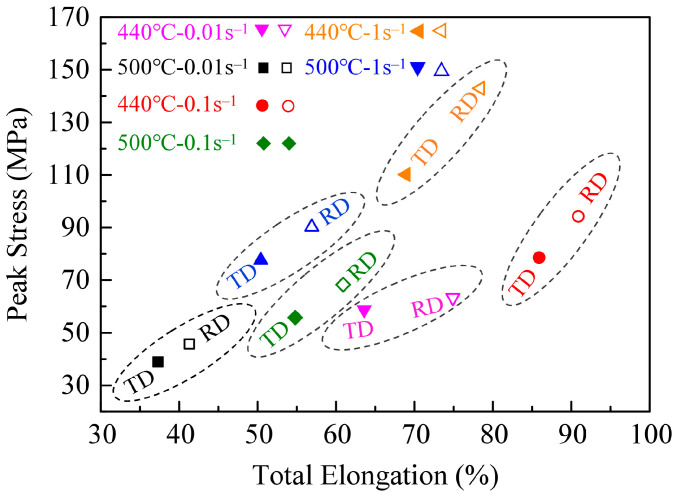
Variation in PS and total EL with deformation temperature, strain rate, and loading direction.

Figure 1 shows the flow curves of 2195 Al–Li alloy samples, which are tested at 440 and 500 °C, and the strain rates of 0.01 s^−1^, 0.1 s^−1^, and 1 s^−1^. It can be seen that the tensile stress first increases with the rising strains during the elastic deformation stage and then decreases after approaching peak stress (PS). Subsequently, with a further increase in strain, the flow stress dramatically decreases until the fracture occurs. Figure 2 displays the variation in PS and total elongation (EL) with the different deformation conditions. Overall, the PS and EL along RD are all larger than the results of TD. And, the effect of the loading direction on stress is greater than that on EL. Under the same deformation condition, the PS values along RD are apparently larger than TD. For the tests carried out at 500 °C-0.01 s^−1^, the PS values of the sheets stretched along the RD and TD are 45.7 MPa and 38.9 MPa, respectively. The difference is more obvious under higher strain rates. The PS is 110.2 MPa of the sheet stretched along TD, and the value of RD reaches 142.9 MPa under 440 °C-1 s^−1^. According to the previous studies [24,25], to better characterize the evolution of PS along RD and TD, the PS anisotropy can be defined as the difference value (D-value) between the PS along the RD and TD divided by the minimum PS value (PSmin), as shown in Equation (1). Similar to the PS, the EL anisotropy can be defined by Equation (2).

(1) $$\mathrm{PS}\;\mathrm{anisotropy}\;={(\mathrm{PS}}_{\mathrm{RD}}-{\;\mathrm{PS}}_{\mathrm{TD}})/\mathrm{PSmin}\times 100\%$$

(2) $$\mathrm{EL}\;\mathrm{anisotropy}\;={(\mathrm{EL}}_{\mathrm{RD}}-{\;\mathrm{EL}}_{\mathrm{TD}})/\mathrm{ELmin}\times 100\%$$

Corresponding PS and EL anisotropy and tensile properties are listed in Table 2. Most of the PS anisotropy results are larger than 15%, and the maximum PS anisotropy reaches 29.7%. All the ELs along RD are a little larger than those of TD, the EL anisotropy is about 10%, and the maximum EL anisotropy is 17.6% (440 °C-0.01 s^−1^). The minimum EL anisotropy also reaches 5.8% (440 °C-0.01 s^−1^). The anisotropy results in the present research are close to or even larger than the results of previous studies, which are carried out at room temperature of the 2195 Al–Li alloy [26]. As a result, it can be easily concluded that the 2195 Al–Li alloy sheet has a significant mechanical property anisotropy under present deformation conditions. 

### 3.2. Fracture Surfaces of Different Deformation Conditions

**Figure 3:**
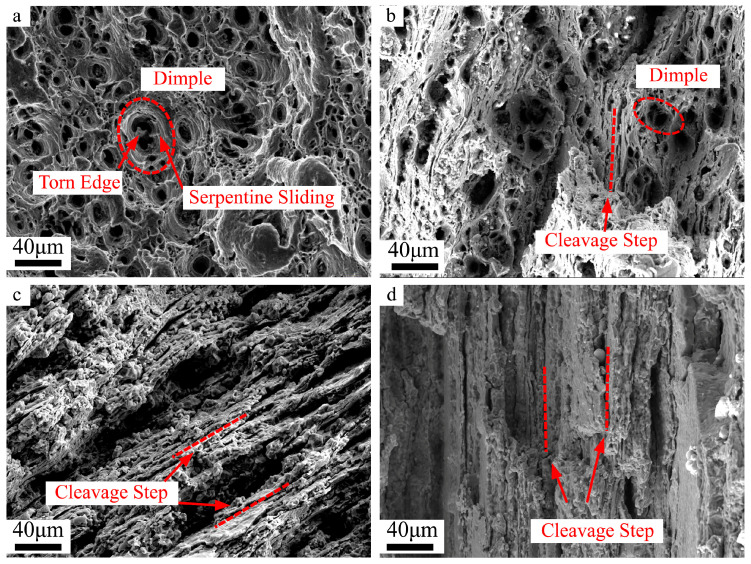
Fracture morphology of different deformation conditions: (**a**) 440 °C-0.1 s^−1^-RD, (**b**) 440 °C-0.1 s^−1^-TD, (**c**) 500 °C-0.1 s^−1^-RD, and (**d**) 500 °C-0.1 s^−1^-TD.

Figure 3 shows the results of the fracture observation at 440 °C and 500 °C at 0.1 s^−1^ along RD and TD. As shown in Figure 3a, the fracture morphology of the samples deformed at 440 °C-0.1 s^−1^ was a typical dimple characteristic with a large number of dimples surrounded by tearing edges appearing on the fracture surface. The dimples in the sample stretched along RD (Figure 3a) are more and deeper than those of TD (Figure 3b), which indicates that the samples stretched along RD experience more apparent plastic deformation, and the EL is also larger [27]. Moreover, a small quantity of cleavage steps can still be found from the fracture surface of the TD sample. The fracture morphology changes from ductile dimples to a large number of cleavage steps, and only a few micropores exist. It indicates an entirely brittle cleavage fracture along RD and TD at 500 °C (Figure 3c,d).

### 3.3. Microstructure Evolution

#### 3.3.1. OM and EBSD Examination for Grain Structure

**Figure 4:**
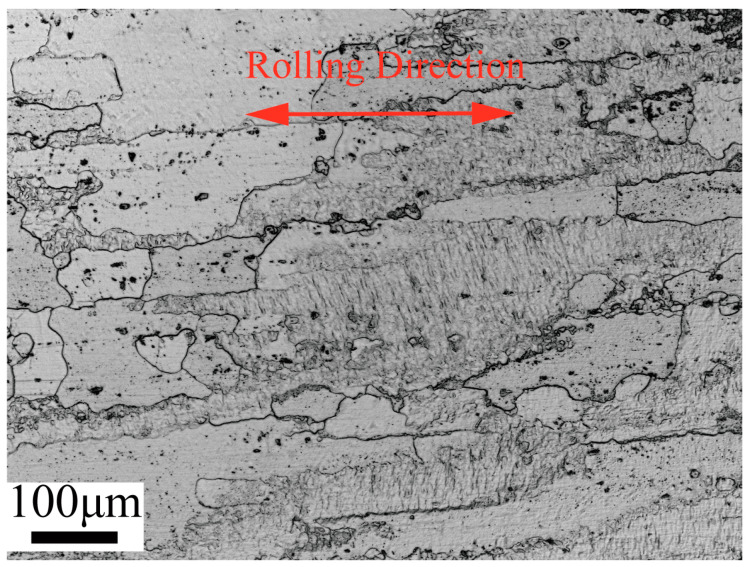
OM image of the 2195 Al–Li alloy initial sheet.

**Figure 5:**
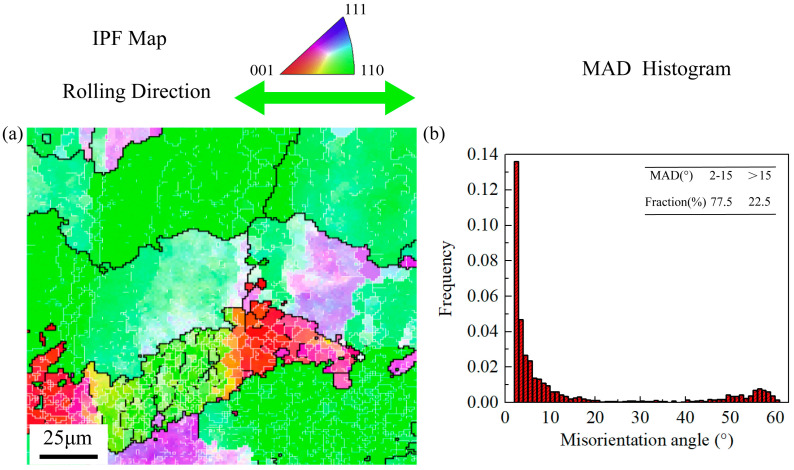
IPF map and MAD histogram of the 2195 Al–Li alloy initial sheet: (**a**) IPF map and (**b**) MAD histogram.

Figure 4 shows the OM image of the initial 2195 Al–Li alloy sheet. Most of the initial coarse grains are elongated along RD and form a bonded structure. Figure 5 displays the EBSD results of the initial sheet. The high angle grain boundaries (HAGBs, misorientation angle > 15°) and the low angle grain boundaries (LAGBs, misorientation angle 2–15°) are represented by the black and white lines, respectively. It can be seen that the fraction of LAGBs achieves 77.5% in the misorientation angle distribution (MAD) map.

**Figure 6:**
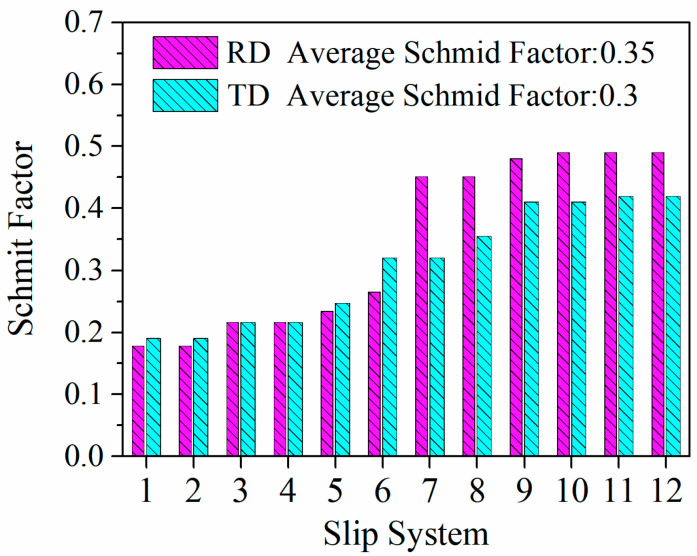
Schmid factor of different slip systems in <101> orientated grains.

With respect to the orientation of the grains in Figure 5, most initial grains have <110> orientation, and only a small amount of <100> orientation and <111> orientation equiaxed grains with small sizes exist. Schmid factor can reflect the hardness of grain slip, i.e., the hardness of plastic deformation, which can be calculated from EBSD data [28,29,30]. The plastic deformation of metals is a complex process involving the interaction of multiple grains. Therefore, the study on the Schmid factor of a single grain in the initial material is meaningless for investigating the hot tensile properties of Al–Li alloy sheets along different loading directions. So, in order to study the relationship between the orientation of the initial sheet and the anisotropy of tensile properties of the cold-rolled sheet, the average Schmid factors of the whole <110> grains stretched along RD and TD were counted, and the statistics results are listed in Table 3 and Table 4. To better compare the Schmid factor of different loading directions, the results are presented in the form of the bar chart in Figure 6. The average Schmid factor along RD is 0.35, and the result of TD is 0.3. At the same time, Table 3 and Table 4 show that the maximum Schmid factor along RD is also larger than TD. Therefore, it can be considered that the deformation along RD is easier to start than TD under hot tensile conditions.

**Figure 7:**
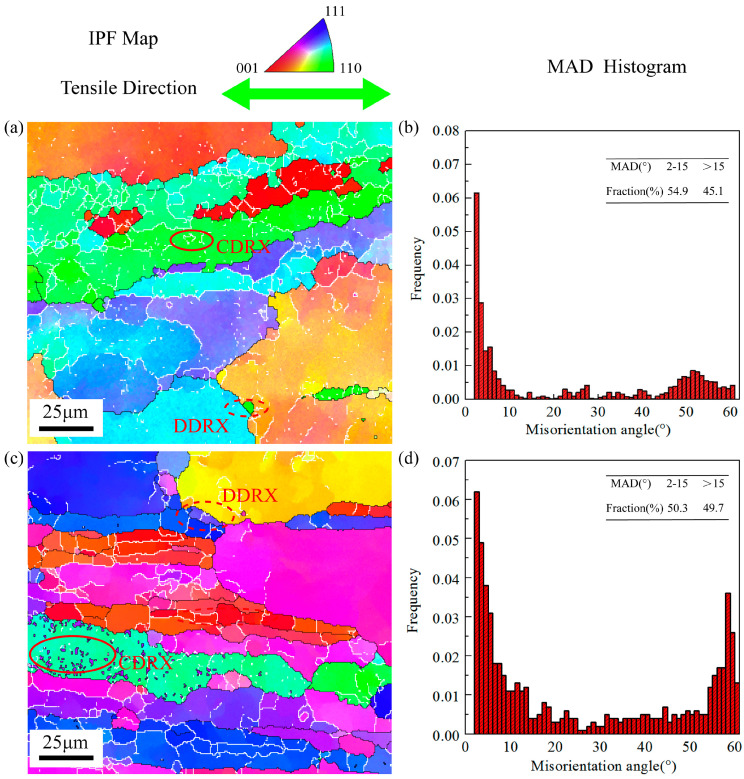
IPF maps, MAD histograms under 440 °C and strain rate 0.1 s^−1^ of strain 0.6 along different directions: (**a**) IPF map along RD, (**b**) MAD histogram along RD, (**c**) IPF map along TD, and (**d**) MAD histogram along TD.

Figure 7 displays the EBSD results of the samples stretched under the deformation conditions of 440 °C-0.1 s^−1^-0.6 (strain) along RD and TD. It can be seen that the grains stretched along RD noticeably elongated along the tensile direction from Figure 7a, and a certain amount of DRX grains occurs in the initial grain boundary position (discontinuous dynamic recrystallization (DDRX)) and inside the initial grains (continuous dynamic recrystallization (CDRX)). Most of the DRX grain boundaries are still LAGBs. The fraction of the HAGBs is 45.1%. Figure 7c displays the EBSD results of the samples stretched along the TD. As can be seen, there are more DRX grains, especially small-sized CDRX grains, within the initial coarse grains compared with RD, and the fraction of HAGBs rises to 49.7%. Similar to RD, the grains of TD also significantly elongate along the tensile direction, and the original banded structure along the rolling direction has disappeared. However, due to the distinction in the shape of initial grains, the grains stretched along TD produced more significant shape changes during the hot tensile process. The influence of the initial grain shape on the dynamic softening mechanism will be discussed later.

**Figure 8:**
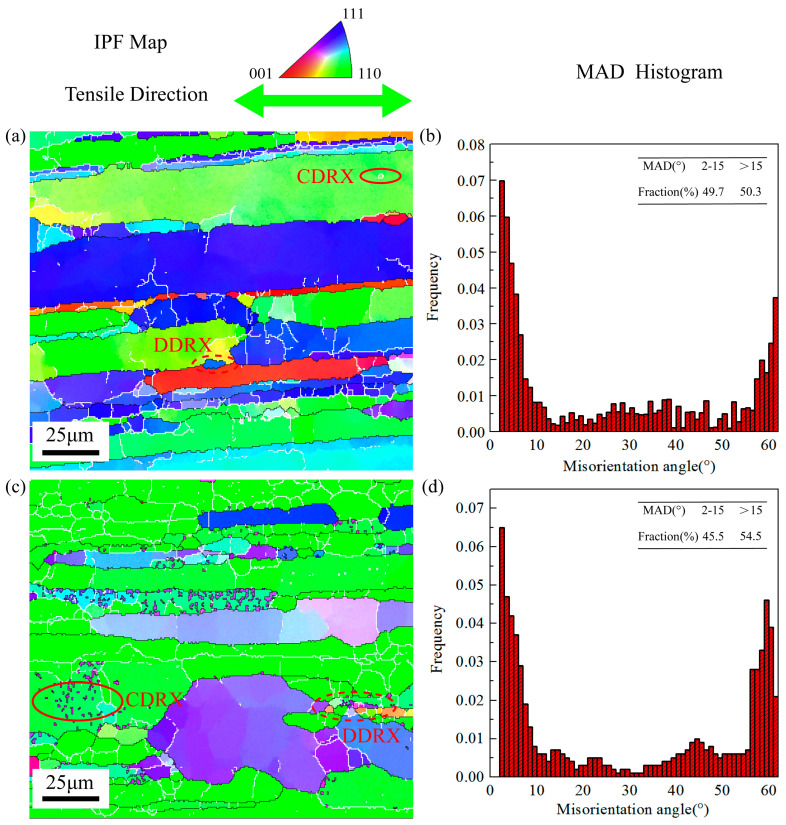
IPF maps, MAD histograms under 500 °C and strain rate 1 s^−1^ of strain 0.35 along different directions: (**a**) IPF map along RD, (**b**) MAD histogram along RD, (**c**) IPF map along TD, and (**d**) MAD histogram along TD.

**Figure 9:**
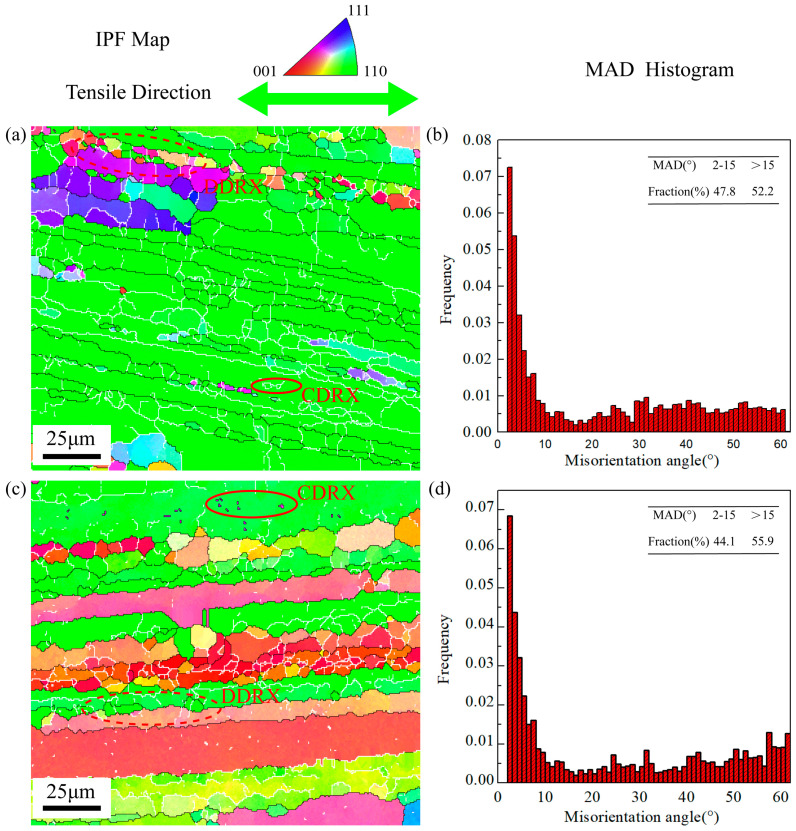
IPF maps, MAD histograms under 500 °C and strain rate 0.1 s^−1^ of strain 0.4 along different directions: (**a**) IPF map along RD, (**b**) MAD histogram along RD, (**c**) IPF map along TD, and (**d**) MAD histogram along TD.

Figure 8a displays the inverse pole figure (IPF) map under the deformation condition of 500 °C-1 s^−1^-0.35 along RD. Due to the higher strain rate, the grains are prominently jagged. There are few CDRX grains and only a certain number of DDRX grains at the boundary of the initial coarse grains. The proportion of HAGBs is 50.3% under this condition. Figure 8c is the IPF map under the same deformation condition along TD. Similar to the result of 440 °C-0.1 s^−1^-0.6, the deformation along TD produces more DRX grains, and the proportion of HAGBs is also higher than RD, which rises to 54.5%. In addition, it can be found that the increase in CDRX grains is more significant. Figure 9a shows the IPF map of samples stretched along RD under the condition of 500 °C-0.1 s^−1^-0.4. Compared with other deformation conditions, the deformation temperature under this condition is relatively higher, and the strain rate is relatively slower. Consequently, there are more DRX grains, and the proportion of HAGBs is 52.2%. Figure 9c is the IPF map of samples stretched along TD. The proportion of HAGBs is also relatively higher, reaching 55.9%. Unlike the EBSD results of TD under the above deformation conditions, the small-sized DRX grains have grown and replaced some of the initial grains. In contrast with the results along TD, a large number of new DRX grains still remain as LAGBs along RD under the same deformation condition.

#### 3.3.2. TEM Examination for Dislocation and Sub-Grain

**Figure 10:**
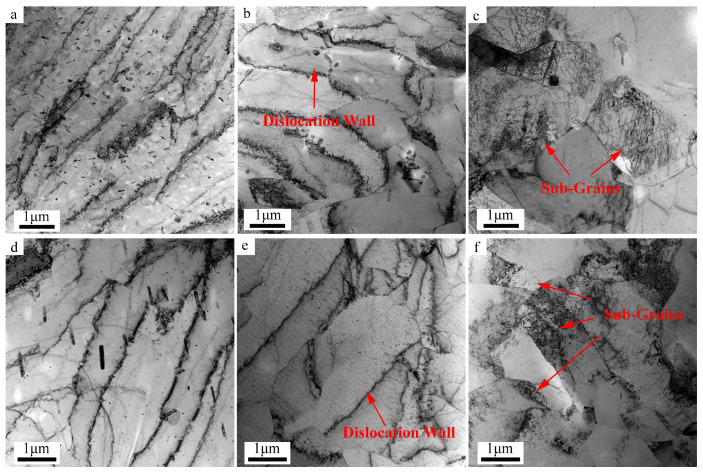
TEM images of microstructure morphologies under different deformation conditions: (**a**) 440 °C-0.1 s^−1^-0.4-RD, (**b**) 440 °C-0.1 s^−1^-0.6-RD, (**c**) 440 °C-0.1 s^−1^-0.65-RD, (**d**) 440 °C-0.1 s^−1-^0.4-TD, (**e**) 440 °C-0.1 s^−1^-0.5-TD, and (**f**) 440 °C-0.1 s^−1^-0.6-TD.

To better characterize the substructure evolution process of the research metal during the hot tensile test, TEM experiments were carried out. Figure 10 shows the TEM images for the specimens stretched to the strains of 0.4, 0.6, and 0.65 under the condition of 440 °C-0.1 s^−1^ along RD and strains of 0.4, 0.5, and 0.6 along TD. As can be seen from the results of the RD, when the strain is 0.4, there are a certain number of dislocation walls and precipitations in the matrix. Moreover, there is a tendency for dislocation walls to aggregate and form dislocation cells, which are shown in Figure 10a. As the hot tensile strain rises to 0.6, the dislocations density starts to decrease, most of the randomly distributed dislocation walls turn into the dislocation cells, the boundaries of the cells start to sharping, and the dislocation density inside dislocation cells obviously decrease due to the migration and annihilation of dislocations. This process of dislocation transformation is a typical feature of sub-grain boundary formation (Figure 10b). As can be seen in Figure 10c, at strain 0.65, the previously formed dislocation cells transform into sub-grains with a large curvature by polygonization in the process of CDRX. The TEM results of TD are similar to those of RD. Dislocation also undergoes the above transformation process with increasing strains.

It is worth noting that, at strain 0.6 along TD, a large number of entanglement dislocations are also found, along with the appearance of multiple sub-grains, indicating that the deformation under this condition along TD is not uniform (Figure 10f).

### 3.4. Discussion

The EBSD results show that the fractions of HAGBs along RD are all larger than TD. Combining the results in IPF maps, it can be seen that the DRX degree of TD is obviously higher than that of RD. Generally, under hot deformation conditions, the most important factor affecting the peak stress of aluminum alloys is the degree of DRX [20,21,30]. Consequently, the higher degree of DRX results in lower PS along TD, although the greater difficulty in opening the slip system at the beginning of the deformation.

**Figure 11:**
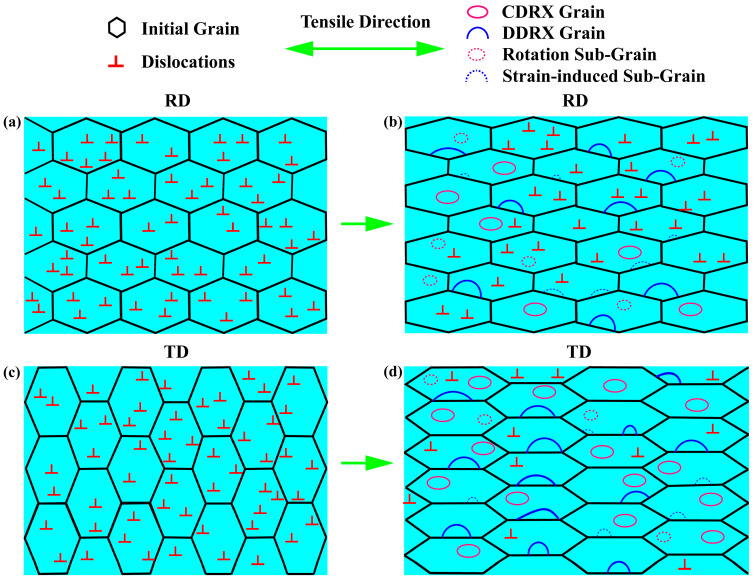
The model of DRX during the process of hot tensile deformation along RD and TD: (**a**) initial grains along RD, (**b**) hot tensile deformation grains along RD, (**c**) initial grains along TD, and (**d**) hot tensile deformation grains along TD.

Figure 11 depicts the model of DRX grain evolution during the process of hot tensile deformation to clarify the differences in the grains evolution along TD and RD. Noticeably the degree of DRX along TD is higher than that along RD, which is concluded from the results of EBSD. In the state of uniaxial tension state, to make the hot tensile easier to perform, the grains will change into an orientation that is more conducive for the slip system to slip by rotation. The average Schmid factor of the initial grains stretched along RD in different slip systems is higher than that of TD. As a result, the deformation along RD is easier to start. For the larger external force required to start the slip system, deformation along TD is more difficult. Therefore, during the deformation process, the grains tend to rotate to an orientation that is more conducive to deformation. In the process of the CDRX, the pre-formed sub-grain boundaries continuously absorb other sub-grain boundaries and dislocations through rotation to become HAGBs, completing the recrystallization nucleation process [20,30]. The grains of the samples stretched along TD tend to rotate more during the deformation process, which will significantly promote the CDRX. Consequently, compared with the deformation along RD, there are more obvious CDRX grains inside the initial grains along TD under the same deformation condition, which can be seen in the IPF maps. The results in TEM show that there is a distinct dynamic recovery (DRV) and CDRX of the 2195 Al–Li alloy during the hot tensile deformation process along RD and TD under present deformation conditions. As the amount of strain increases, the dislocation organization continues to evolve, forming dislocation cells and transforming them into sub-grains. This result supports the preceding discussion on grain DRX in the EBSD part.

In addition, according to the shape of the sample after tensile deformation, it can be easily concluded that when the samples are stretched along RD and TD, the grains are prominently elongated along the loading direction. When the samples deform along RD, the original shape of the grains is elongated along the rolling direction, and the loading direction is consistent with the initial grain elongation direction. However, the loading direction is perpendicular to the initial grain elongation direction when the samples are stretched along TD, and the IPF maps also show that the elongated direction of the grain will naturally transform into the perpendicular direction. According to the previous studies [31], under the same tensile strain, the grain boundary migration will contribute more to the deformation when the samples are stretched along TD than along RD because of the larger change in grain shape. Thus, stretching along TD advances the local migration of the grain boundaries, leading to an easier bulging of initial boundaries. As the deformation processes further, more strain-induced sub-grain boundaries would separate the bulging parts from the original grains, and the DDRX, which is characterized by the local bulge of the boundaries, will be promoted obviously. Meanwhile, the higher tendency of the sub-grain rotation and the grain boundary migration will also promote grain growth of DDRX and CDRX in addition to nucleation. Therefore, under the same deformation conditions, the DRX degree of loading direction along TD is distinctly higher, and a higher proportion of HAGBs is present.

Generally, the thermo-plasticity of aluminum alloys is consistent with the degree of DRX. The higher the DRX degree is, the better the thermo-plasticity is, as adequate DRX is advanced for migrating grain and sub-grain boundaries and isolating the tensile cracks [21,32]. The experimental results in this study contradict this rule. As mentioned above, when the samples are stretched along RD, the higher average Schmid factor will result in a simpler start of deformation and better hot ductility. Therefore, it indicates that, compared with the degree of recrystallization, the Schmid factor and banded structure formed after rolling have a greater impact on the thermo-plasticity of the 2195 Al–Li alloy in actual hot deformation.

## 4. Conclusions

In the present research, the anisotropic mechanical behaviors and microstructure evolution of 2195 Al–Li alloy sheets were investigated under the strain rate of 0.01, 0.1, and 1 s^−1^ and the temperature of 440 and 500 °C. The grain dynamic softening mechanism along different loading directions was studied deeply. The following conclusions are summarized:

(1) The PS and EL along the RD are all larger than the results of TD, and the anisotropy in PS is more significant than EL. Most of the PS anisotropy values are larger than 15%. For the tests carried out at 440 °C^−1^-1 s^−1^, the PS values of the sheets stretched along the RD and TD are 142.9 MPa and 110.2 MPa, respectively. For the EL anisotropy, all the values are about 10%.
(2) The fracture surface morphology is typical ductile characteristics at 440 °C. The dimples of the sample stretched along RD are more and deeper than those of TD. At 500 °C, the fracture mode turns into an entirely brittle cleavage fracture along RD and TD.
(3) Most initial grains are <101> orientation, and few <100> and <111> orientation grains exist. The average Schmid factor along RD and TD is 0.35 and 0.30, and the maximum Schmid factor along RD is also larger than TD; therefore, the deformation along RD is easier than along TD.
(4) The fractions of HAGBs along TD are all about 5% larger than those of RD, and there are more small-sized CDRX grains inside the initial grains and more DDRX grains at the initial grain boundaries along TD. This is caused by the difference in the Schmid factor and the initial grain shape along RD and TD. A model of grain DRX during the hot tensile deformation process is developed. Meanwhile, the effect of the Schmid factor and bonded structure formed after rolling on the hot ductility is more prominent, thus presenting a higher EL and a better hot ductility for the deformation along RD.

## Figures and Tables

**Table 1:** The chemical element compositions of the initial 2195 Al–Li alloy rolled sheet (wt %).

Ele.	Cu	Li	Mg	Ag	Zr	Fe	Al
wt %	4.1	1.01	0.43	0.4	0.11	0.05	Bal.

**Table 2:** Tensile property values of the 2195 Al–Li alloy with different deformation conditions.

Deformation Condition	Loading Direction	PS/MPa	PS Anisotropy/%	EL/%	EL Anisotropy/%
440 °C-0.01 s^−1^	RD	63.3 ± 2	7.8	74.8 ± 2	17.6
	TD	58.7 ± 2		63.5 ± 2	
440 °C-0.1 s^−1^	RD	94.2 ± 3	19.9	90.9 ± 3	5.8
	TD	78.6 ± 2		85.9 ± 3	
440 °C-1 s^−1^	RD	142.9 ± 5	29.7	78.4 ± 2	13.8
	TD	110.2 ± 4		68.8 ± 2	
500 °C-0.01 s^−1^	RD	45.7 ± 1	17.3	41.3 ± 1	10.8
	TD	38.9 ± 1		37.3 ± 1	
500 °C-0.1 s^−1^	RD	68.2 ± 2	22.4	60.9 ± 2	11.2
	TD	55.7 ± 2		54.8 ± 2	
500 °C-1 s^−1^	RD	90.2 ± 3	16.2	56.9 ± 2	13.1

**Table 3:** Schmid factor of slip systems in <101> orientated grains, which are stretched along RD.

Slip Plane	Slip System	Schmid Factor
	(111) [1–10]	0.451
[111]	(111) [10–1]	0.178
	(111) [01–1]	0.234
	(−111) [110]	0.216
[−111]	(−111) [101]	0.49
	(−111) [01–1]	0.265
	(111) [110]	0.216
[1–11]	(111) [10–1]	0.178
	(111) [011]	0.49
	(111) [1–10]	0.451
[11–1]	(111) [101]	0.49
	(111) [011]	0.48

**Table 4:** Schmid factor of slip systems in <101> orientated grains, which are stretched along TD.

Slip Plane	Slip System	Schmid Factor
	(111) [1–10]	0.410
[111]	(111) [10–1]	0.216
	(111) [01–1]	0.355
	(−111) [110]	0.419
[−111]	(−111) [101]	0.19
	(−111) [01–1]	0.247
	(111) [110]	0.419
[1–11]	(111) [10–1]	0.216
	(111) [011]	0.32
	(111) [1–10]	0.41
[11–1]	(111) [101]	0.19
	(111) [011]	0.32

## Data Availability

Not applicable.
